# Functional results after carpal tunnel release in mucopolysaccharidosis

**DOI:** 10.1186/s13023-021-01982-3

**Published:** 2021-09-09

**Authors:** Giana Silveira Giostri, Camila Deneka Arantes Souza, Alencar Kenji Nagai, Mara Lucia Schmitz Ferreira Santos, José Silvany Pacheco Sampaio, Flavia David João de Masi Nassif

**Affiliations:** 1 Serviço de Cirurgia da Mão do Hospital Pequeno Príncipe, Curitiba, PR Brazil; 2 Serviço de Neurologia Pediátrica do Hospital Pequeno Príncipe, Curitiba, PR Brazil; 3 Hospital Universitário Cajuru, Curitiba, PR Brazil; 4 PUC-PR, Curitiba, PR Brazil; 5 Ambulatório Ortopedia Pediátrica, Rua Desembargador Motta, 1070 - Água Verde,, Curitiba, PR 80250-060 Brazil

**Keywords:** Mucopolysaccharidosis, Carpal tunnel syndrome, Median nerve, Nerve compression

## Abstract

**Background:**

Mucopolysaccharidosis consists of a group of diseases caused by the deficiency of lysosomal enzymes, which may lead to the compression of the median nerve in the carpal tunnel due to the accumulation of glycosaminoglycan, resulting in the hand disability. The study purpose is to present functional results of carpal tunnel release in mucopolysaccharidosis patients. Patients were selected from an enzyme replacement group in the Department of Pediatric Neurology. The legal guardians of the patients were informed about the likely functional change of the hands induced by compression of the median nerve. Clinical evaluation was performed in those patients who received their legal guardians’ consent to participate and was included inspection, assessment of functional level, wrinkle test and the digital pinch function to manipulate small and large objects. Ultrasound and electromyography were performed to confirm the clinical median nerve compression. Bilateral extended opening technique was performed to access the carpal tunnel and analyze the anatomic findings of the median nerve and the flexed tendons of the fingers. After the surgical release of the carpal tunnel, the clinical evaluation was repeated. Subjective observations of the legal guardians were also considered.

**Results:**

Seven patients underwent bilateral surgical opening of the carpal tunnel; six boys, mean age of 9.5 (5 to 13), five of them presenting Type II mucopolysaccharidosis, 1 Type I and 1 Type VI. The average follow-up was 12 months (10–13 months). The functional results observed included the improvement in the handling of small and large objects in all children who underwent decompression of the median nerve. The comparison between the pre-operative and post-operative functional levels revealed that 2 patients evolved from Level II to IV, 3 from Level III to IV, 1 from Level IV to V and 1 patient remained in Level III. Tenosynovitis around the flexor tendons and severe compression of the median nerve in the fourteen carpal tunnels were observed during the surgical procedure. In 6 wrists, partial tenosynovitis was performed.

**Conclusions:**

Despite the improvement in the overall function of the children' hands, we cannot conclude that only surgery was responsible for the benefit. Better designed studies are required

## Background

Mucopolysaccharidoses (MPS) are caused by the deficiency of lysosomal enzymes resulting in the intracellular accumulation of semi degraded compounds derived from lysosomal deposition (glycosaminoglycans) that characterize the type of disease: I—Hurley Syndrome; II—Hunter Syndrome; III—Sanfilippo Syndrome; IV—Mórquio Syndrome; V—Scheie Syndrome; VI—Maroteaux-Lamy Syndrome; VII—Sly Syndrome; IX—Natowicz disease (Table 1) [1–4]. The accumulation of glycosaminoglycans (GAGs), mainly heparan and dermatan sulfates, lead to the thickening of the flexor retinaculum [1, 3, 5, 6] and consequent compression of the median nerve [3].

**Table 1:** Classification of the mucopolysaccharidoses

Name of the syndrome	GAG substrates
MPS I	Dermatan sulphate
Hurley, Hurley-Scheie and Scheie syndromes	Heparan sulphate
MPS II	Dermatan sulphate
Hunter syndrome	Heparan sulphate
MPS III	Heparan sulphate
Sanfilippo syndrome	
MPS IV	Keratan sulphate
Morquio syndrome	
MPS VI	Dermatan sulphate
Marateaux-Lamy syndrome	
MPS VII	Dermatan sulphate
Sly syndrome	Heparan sulphate
MPS IX	Hyaluronan
Natowicz disease	

The overall MPS incidence is 1 in 25,000 live births [1]. It is usually bilateral and may be associated with Carpal Tunnel Syndrome (CTS), tenosynovitis and the trigger finger condition [7, 8].

The association with CTS has been reported in 90% of the MPS type I and II and rarely observed in type III [3]. Children with MPS may present early compression of the median nerves usually before the age of five [3]. Nocturnal paresthesia, positive Tinel and Phalen signal, the classic clinical features observed in adults, are not observed in children with MPS [3, 9]. Although symptoms, such as atrophy of the digital pulp, reduction of sweating, atrophy of the tendon muscles, awkward position of the fingers and decreased sensitivity, may be present, the diagnosis can frequently represent a challenge, considering the characteristic disorders of the disease, which include aggressiveness and cognitive difficulties [1, 3, 5, 10]. Delay in identifying these clinical findings results in a progressive decrease in the hand function due to constant and unceasing compression of the median nerve in the carpal tunnel [11, 12].

The atypical clinical incidence of CTS, associated with the lack of expression of symptoms in children with relevant intellectual disability, increases the importance of an early screening and diagnosis of the median nerve compression in patients with MPS. Haddad et al. [5] have recommended early attention regarding the difficulty in using the thumb to manipulate objects and the increased sweating in the median nerve innervated region. According to these authors, the clinical conditions develop before the more evident signs, such as the thenar hypotrophy and digital pulp hypotrophy, can be identified.

To anticipate the CTS diagnosis, Van Heest et al. [13, 14] advised that electrodiagnostic testing be initiated as a screening tool by the age of 3 in an effort to identify the disease at an early stage or establish a baseline for later comparison in a new exam [15]. They also recommended electrodiagnostic testing to rule out other potential pathologies including cervical radiculopathy, peripheral polyneuropathy and brachial plexopathy.

Conversely, ultrasound (US) to evaluate the carpal tunnel syndrome in children with MPS is a convenient diagnostic method. It is less expensive and pain free. According to Baumer et al. and Jester [16, 17], this diagnostic technique seems to provide higher sensitivity regarding the changes in the median nerve when compared to electrophysiological examinations. The difficulties reported include little collaboration by the pediatric patient and the examiner-dependent nature of the US. Therefore, the earlier use of US as a screening exam, whenever possible, could help in the earlier diagnosis and treatment of the median nerve in the carpal tunnel.

Unfortunately, enzyme replacement therapy and bone marrow transplant for MPS do not prevent the development of CTS in children. The direct damages to the nerve or its myelin sheath due to glycosaminoglycan deposition have not been identified to date [5, 18]. The presence of these deposits in the synovium of the flexor tendons, along with the thickening of the transverse carpus ligament, contribute to worsen the median nerve compression [10]. Studies have reported bone abnormalities in the carpal tunnel as part of the disease, which may also cause lesions of the median nerve [3]. Fisher et al. and Yuen et al. [11, 12] recommend early carpal tunnel release to prevent permanent injury in median nerve; however, there is no determined age limit to perform the procedure.

In view of all these considerations, our objective is to evaluate the functional results of the carpal tunnel release in a cohort of children with MPS.

## Methods

This prospective clinical cohort study includes MPS patients from an enzyme replacement group at the Department of Pediatric Neurology. The diagnostic methods used included urine glycosaminoglycan levels, enzymology and gene sequencing. The medication used to treat MPS II was Idursulfase (Elaprase) at a dose of 0.5 mg/kg administered once a week intravenously. In the treatment of MPS I, we use Laronidase (Aldurazyme) at a dose of 0,58 mg/kg intravenously weekly and in MPS VI, Galsulfase (Naglazyme) 1 mg/kg was used intravenously once a week [19]. The patients also underwent speech therapy, occupational therapy and received psychological support from a multidisciplinary team. The researchers visited the patients during the enzyme administration for 3 months. In these informal contacts, the legal guardians of the children were asked to report on possible alterations in the patients’ hand function, mainly in the thumb pinch. Additionally, information was provided about the part of the child’s hand that was used to turn a magazine or a book page. The likelihood of the nerve compression was addressed; however, only 7 from the legal guardians agreed to participate voluntarily and signed the consent form, approved by the Ethics Research Committee (CAAE: 02277012.6.0000.0097).

The selected cohort of patients was evaluated clinically to confirm the bilateral MPS diagnosis. Ultrasound (US) and Electroneuromyography (EMG) were performed for the studied cases. Pre and postoperative clinical evaluation of both hands consisted of inspection (thenar and hypothenar trophism, digital position), digital pinch function to manipulate small and large objects, assessment of functional level (AFL) [3] and wrinkle test. Further analysis included the observations as to whether the pinch was performed with the thumb and forefinger or with the minimum or annular fingers. The handling of large and small objects and the accomplishment of tasks such as to button a shirt, close a zipper, flip a book page, and use scissors defined the level of hand function (AFL showed in Table 2).

**Table 2:** Assessment of functional level (AFL) [3]

Functional classification (AFL)	Assessed manual tasks
1	Performs just passive movements
2	Grabs large items
3	Grabs small objects, perform the pinch
4	Flips a page, uses the telephone
5	Buttons a shirt, closes a zipper, writes or uses scissors

**Fig. 1:**
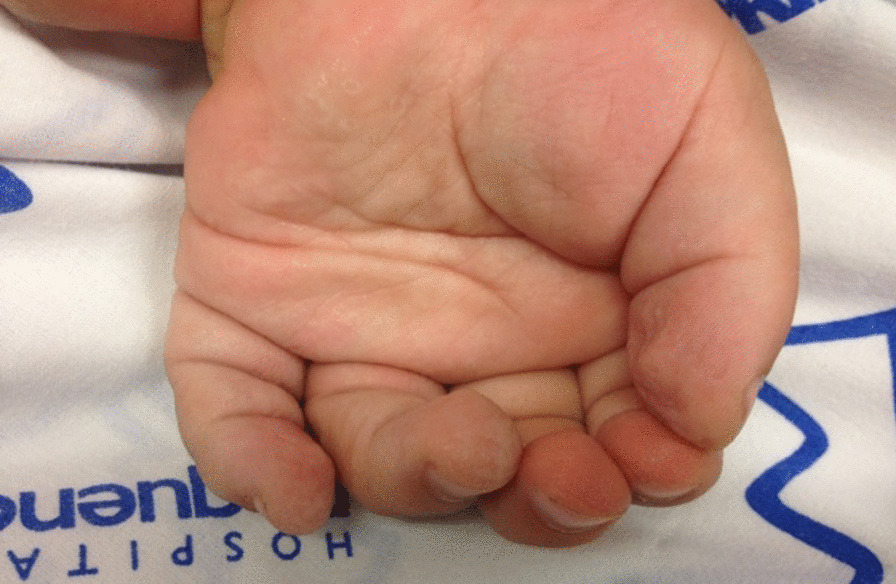
Wrinkle test. Wrinkling on the thumb digital pulp and on the ring and little fingers after water immersion is observed

Wrinkle test was performed by immersing the hands in warm water for 15–20 min. Wrinkles in digital pulp evaluation were observed on normal hands. Result was considered positive when at least one of the fingers innervated by the median nerve developed wrinkles during the test. Conversely, the result was negative when none of the fingers innervated by the median nerve presented wrinkles. Figure 1 shows wrinkling on the thumb digital pulp and on the ring and little fingers after the immersion, and absence of wrinkles on the forefinger and on the middle finger.

### Surgical technique description

The extended surgical technique to release the transverse carpus ligament was applied on both hands at the same time. A long longitudinal and curvilinear incision was made between the thenar and hypothenar muscles, ulnar to the flexor tendons and medial towards the medial cutaneous palmar branch nerve. Flexor tendons and median nerve were assessed after the carpal tunnel was opened and excessive tenosynovia around the flexor tendons was removed. Tourniquet was released and the flexor tendons and median nerve were re-evaluated. Skin closure was performed with nonabsorbable suture followed by wound dressing.

## Results

Six of the 7 included patients were male, mean age of 9 years and 5 months (ranging from 4 to 12 years old). Table 3 shows the characteristics of the studied patients. Five cases corresponded to MPS type II (Hunter), 1 type I (Hurley) and 1 type VI (Maroteaux-Lamy). ERT was performed with an average of 18 months (minimum 13 months and maximum 22 months) before performing the decompression surgery.

**Table 3:** Characteristics of the sample and results of the 7 cases: 1* Type of MPS: I—Hurley Syndrome, II—Hunter Syndrome, III—Sanfilippo Syndrome, IV—Mórquio Syndrome, V—Scheie Syndrome, VI—Maroteaux-Lamy Syndrome; 2* age in years at surgery; 3* Complementary Exams performed before the surgery. EMG = electromyography. US = Ultrasound; 4* AFL = Assessment of the Functional Level: 5* Negative = 0, Positive = 1. Assessment after 12 month of surgery (mean)

Patient	MPS type (1)	Age at surgery (2)	Exams (3)	AFL Preop (4)	Preop wrinkle test (5)	Follow-up (6)	AFL Postop (4)	Postop wrinkle test (5)
1	I	4.58	EMG/US	II	0	13	IV	1
2	II	12.8	EMG/US	III	1	13	IV	1
3	IV	11.4	EMG/US	II	0	12	IV	1
4	II	8.17	US	III	0	11	III	0
5	II	9.75	US	III	0	10	IV	1
6	II	5.92	–	III	0	13	IV	1
7	II	12	–	IV	1	12	V	1

**Fig. 2:**
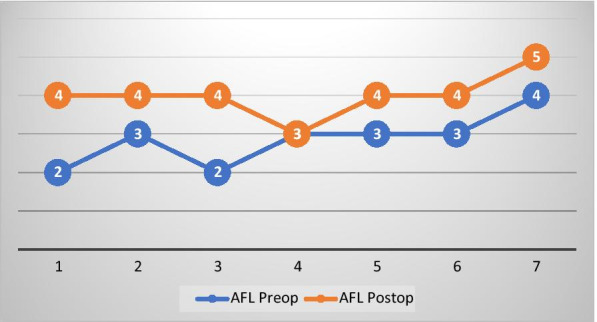
Compared results at preoperative and postoperative assessment of functional level (AFL)

Postoperative results were assessed after an average period of 12 months (ranging from 10 to 13 months). Significant improvement in the handling of small and large objects was observed and the pinch between the thumb and the forefinger was restored, that is, they used their radial fingers, no longer the ulnar margins of the hands. The ability to perform some tasks such as button a shirt, close a zipper, flip a book page and use scissors was also improved, revealing an increase in the functional level of the hands in 6 of the 7 children. However, the thenar hypotrophy, the digital pulp hypotrophy and the flexed position of fingers, remained unchanged. Figure 2 compares pre and postoperative results according to AFL. Two patients evolved from Level II to IV, 3 from Level III to IV, 1 from Level IV to V and 1 remained in Level III.

Two patients with grade II AFL function began to handle small objects using the clamp between the thumb and the forefinger, and transfer them from one hand to the other. Of the 3 Grade III patients, one was able to turn a page using the index fingers, previously done with his little fingers. All legal guardians reported improvement in the children’s daily use of the hands after the carpal tunnel release. They also reported reduced flexion of the fingers, possibly because of the improved flexor tendon excursion after the surgical release.

Preoperatively, the wrinkle test was positive in 4 hands and negative in 10 ones. After the carpal tunnel release, 8 of these 10 hands revealed wrinkling. Four hands maintained their positive results, and the last 2 hands continued showing the negative results at the wrinkle test.

**Fig. 3:**
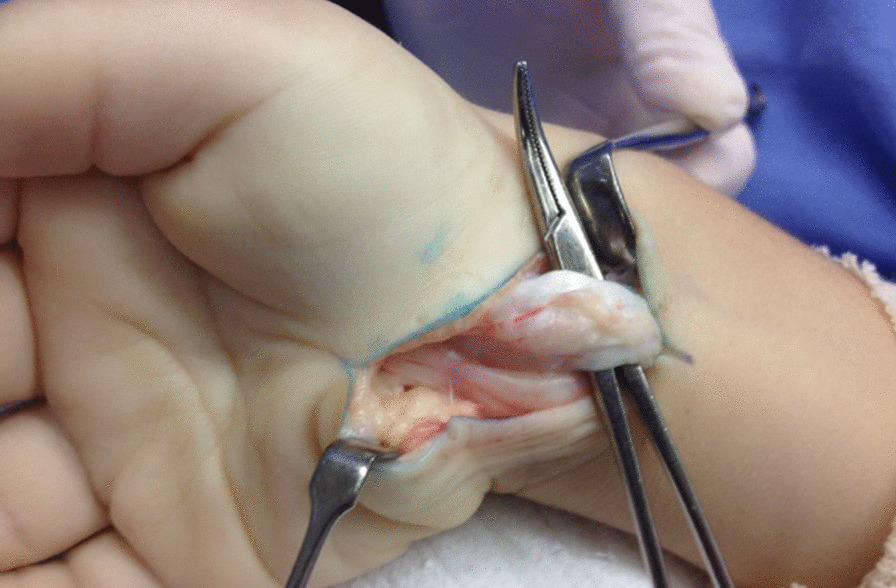
Exposure of the carpal tunnel, with tenosynovitis affecting the flexor tendons

**Fig. 4:**
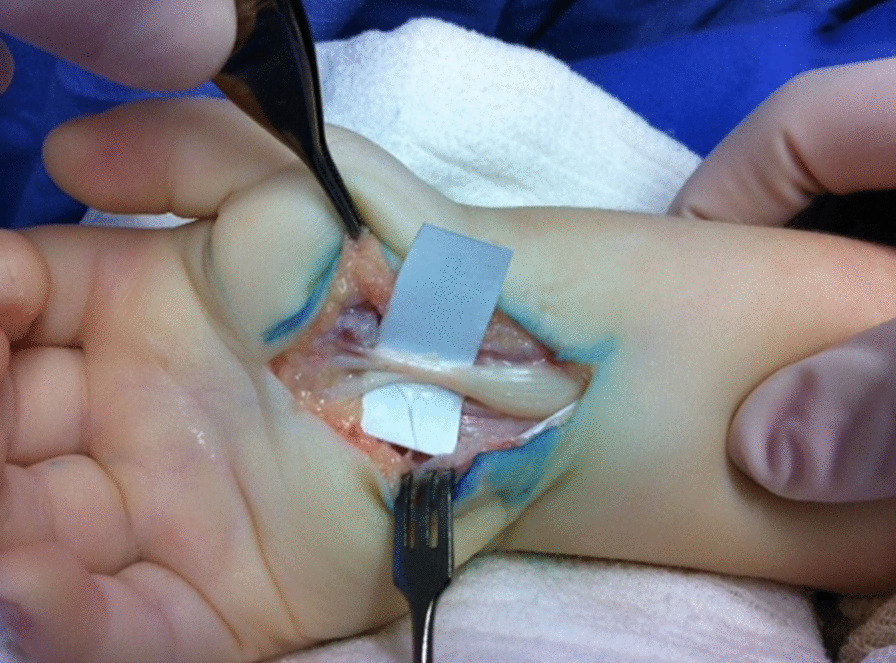
Opening of the carpal tunnel, revealing a significant median nerve compression with hourglass-like constriction

Macroscopic signs of flexor tenosynovitis were observed in 14 carpal tunnel releases. Partial tenosynovectomy was performed in 6 hands to remove the excessive thickening of the synovium among the flexor tendons (Fig. 3). All cases presented significant compression of the median nerve with the development of an hourglass-like constriction (Fig. 4). After the release of the tourniquet, petechiae were observed in the median nerve along the carpal tunnel, revealing nerve damage.

Although US and EMG were recommended in all 7 children, only 5 underwent US and 3 EMG. Two parents did not consent to having their children undergo complementary exams, although consented to surgery.

Four of the 7 legal guardians reported complete satisfaction after their children’s surgical procedure. One of them did not perceive any improvement on the operated hands and 2 reported a worsening condition due to the presence of cicatricial retraction, regardless of the improvement perceived during their hand daily activities.

## Discussion

This study includes the functional results of the hands after the release of the carpal tunnel in a small cohort of children, mean age of 9.2, most of them diagnosed with type II MPS. Jadhav et al. [3] also reported prevalence of CTS in type II MPS in 90% of the assessed cases.

Precise and detailed preoperative evaluation was decisive for the surgical indication in the present study. The ability to perform simple tasks, from grabbing large objects to buttoning a shirt, closing a zipper and using scissors, helped categorize the level of hand function and most importantly helped convey the importance of a surgical intervention to MPS children’s parents.

In order to obtain an early diagnosis of CTS in MPS, Haddad et al. have idealized the Assessment Functional Level (AFL), based on the different levels of difficulty to perform the tasks. The AFL evaluation that we performed showed improvement of the functional level in 6 of the 7 children, in both hands. After the surgery, the children regained their ability to use the radial fingers to handle objects and flip book pages with more frequency. Two parents, whose children’s functional level (AFL III) remained the same after the surgery, complained about the cicatricial retraction in the operated wrists. Nevertheless, these parents emphasized that, despite the aesthetic complaint, the children no longer used the ulnar border to turn pages of books. In this regard, Haddad and colleagues reported improved AFL hand function in all their patients after surgery, particularly in those with type II MPS [5]. Unlike the results reported by the authors, the two patients of the present study, who remained in the Level III AFL, were diagnosed with Type II MPS. Two patients, MPS Type I and Type IV respectively, improved their hand functional level from II to IV.

The mean age of 9.5 of the studied cohort has been considered high for a carpal tunnel release procedure in MPS children, and along with the acute compression of the median nerve observed during the surgery, may account for the lack of improvement in the thenar and digital pulp hypotrophy, after a 12-month follow-up. These conditions could also account for the flexed position of the fingers, despite the improvement observed in the flexion movement of the fingers after the surgery.

Patients with suggestive signs of median nerve compression, with progressive muscle atrophy and gradual loss of hand function, or those with positive results from diagnostic and evaluation exams, are recommended for surgical release of the carpal tunnel to prevent the evolution of the nerve compression, which may cause hand functional limitations and neuropathic pain [3, 6]. Our management and results do not differ from the guidelines however the expressive compression of the median nerve at carpal tunnel release (Fig. 4—Opening of the carpal tunnel, revealing a significant median nerve compression with hourglass-like constriction.) found in all cases of our series contributes to alert the attending physician to check the hand function of these children during the clinical treatment of MPS. To prevent the worsening of the clinical damage, we did not consider neurolysis, even after the release of the tourniquet, as we identified petechiae on the nerve. Viskochil et al. suggested the utilization of urine GAGs with serial clinical surveillance of the patients after the surgery, for possible repeated signs or symptoms derived from the nerve compression [20]. To reduce tension on the nerve, we also suggest partial synovectomy to remove excessive synovial tissue around the flexor tendons. It was performed in 3 patients of our study (6 wrists). Maurer et al. [21] recommended neurolysis of the median nerve, in addition to decompression. Nevertheless, we are in agreement with a recent comparative study showed no additional long term benefit from neurolysis in MPS carpal tunnel release [22].

A careful clinical evaluation of patients with MPS taking into account ectoscopy and functional assessment of the hands in addition to the fact that EMG is associated with false-positive and false-negative results [23] leads us to conclude that EMG is not mandatory to establish the diagnosis [6] of median nerve compression in children with MPS.

We propose that the standardization of EMG in all MPS patients with suspected CTS should not be followed due to the invasive, painful and uncomfortable nature of this procedure for these children [9, 13]. Although US and EMG were recommended by the researchers at the pre-operative stage, only three parents agreed to have their children undergo EMG, and the legal guardians of two children did not consent to any of these diagnostic procedures. Their claim was that the procedures, mainly EMG, was invasive.

The main limitations of this study include a small sample of patients, due to the lack of understanding by the parents or legal guardians of the need of a surgical procedure in MPS patients diagnosed with CTS. Another complicating factor was that few parents consented to clinical examinations, claiming that their children did not complain of any pain on the hands, and hence, refused to allow them to participate in the study. Unfortunately, diagnosis of CTS is often postponed even after muscle thenar wasting and apparent loss of function are observed [23].

This manuscript is our preliminary study we used as a drawing for our current study, which used a more rigorous methodology enhanced with better controls, larger sample size and quality outcome measures reported by the patient (family) in order to define a treatment protocol and its results.

Considering the acute compression of the median nerve revealed in the surgical release procedures in our cohort of patients with a high age mean, we recommend a multidisciplinary management approach, which includes vigilant monitoring of the hand functions, serial clinical exams, and early involvement of a hand specialist in the comprehensive treatment protocols for MPS children diagnosed with CTS.

## Conclusion

Despite the intense nerve compression found in the cases studied and the improvement in the overall function of the children' hands, we cannot conclude that only surgery was responsible for the benefit. Better designed studies are required to be able to demonstrate the real utility of surgical management of CTS.

## Data Availability

All data generated and analysed during this study are included in this published article.

## Abbreviations

MPS: Mucopolysaccharidoses
GAGs: Glycosaminoglycans
CTS: Carpal Tunnel Syndrome
US: Ultrasound
EMG: Electroneuromyography
AFL: Assessment of functional level
